# Considerations for lifetime management of aortic stenosis: Transcatheter aortic valve replacement, surgical aortic valve replacement, and timing of therapy

**DOI:** 10.1016/j.xjse.2024.100028

**Published:** 2024-09-21

**Authors:** Rodrigo Petersen Saadi, Ana Paula Tagliari, Syed Zaid, Gilbert H.L. Tang

**Affiliations:** a Universidade Federal do Rio Grande do Sul, Porto Alegre, Brazil; b Cardiovascular Surgery Department, Hospital Mãe de Deus, Porto Alegre, Brazil; c Cardiology Department, Section of Cardiology, Baylor College of Medicine, Michael E DeBakey VA Medical Center, Houston, Tex; d Department of Cardiovascular Surgery, Mount Sinai Health System, New York, NY

**Keywords:** aortic stenosis, TAVR, SAVR, valve durability, patient-specific treatment, lifetime management

**Figure:**
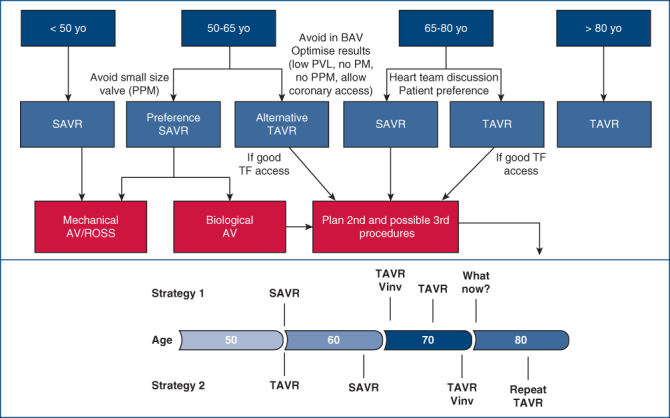
Decision making flowchart for aortic stenosis treatments different age groups. *YO*, Year-old; *SAVR*, surgical aortic valve replacement; *TAVR*, transcatheter aortic valve replacement; *AV*, aortic valve; *VinV*, valve-in-valve; *PPM*, patient–prosthesis mismatch; *BAV*, bicuspid aortic valve; *PVL*, paravalvular leak; *PM*, pacemaker; *TF*, transfemoral.

**Central Message**

Treating severe symptomatic aortic stenosis requires carefully balancing TAVR and SAVR options based on patient profiles and evolving technologies to achieve optimal lifetime outcomes.

Severe aortic valve stenosis (AS) is the primary reason for heart valve intervention in Europe and the United States,^1^ affecting 52.5 individuals (95% confidence interval [CI], 49.4-55.8) per 100,000 patient-years.^2^ The management of AS has evolved dramatically in recent years driven by advancements in treatment modalities and a deeper understanding of disease progression and its impact on survival and quality of life. While elderly, frail patients with a high burden of comorbidities were previously considered inoperable or at high surgical risk for surgical aortic valve replacement (SAVR), the landscape of AS management has been revolutionized by the introduction of transcatheter aortic valve replacement (TAVR).^3^

Over the last decade, TAVR has evolved from an experimental procedure to a treatment option that has demonstrated superiority or at least noninferiority compared to SAVR across all surgical risks.^4^^,^^5^ The decision between SAVR and TAVR should consider not only surgical risk and patient age, but also a range of clinical, anatomic, and procedural factors. These include the feasibility of a transfemoral approach, risk of requiring a pacemaker, and potential procedural complications. Another critical consideration is the risk of structural and nonstructural valve degeneration, which influences the likelihood of future reinterventions and necessitates balancing transcatheter heart valve (THV) durability with the patient's life expectancy. Consequently, such factors as the risk of coronary obstruction, limitations in coronary reaccess, and the feasibility of leaflet modification techniques must be carefully evaluated. These factors impact both short- and long-term outcomes and have critical roles in the decision making process.

This article explores current considerations in the lifetime management of AS, highlighting evolving treatment paradigms and emphasizing a patient-centered approach.

## Timing of Intervention

AS is characterized by progressive thickening, fibrosis, and calcification of the valve leaflets, leading to valve flow restriction, increased left ventricular (LV) afterload, and a consequent hypertrophic response to maintain cardiac output. However, as stenosis progresses, this hypertrophic response eventually decompensates, resulting in symptom development, heart failure, and death.^6^

Despite the incorporation of various drugs in heart failure guideline–directed medical therapy, no medications have been proven to slow or reverse AS progression. Pharmacologic management remains a palliative approach for patients with a life expectancy of <1 year.

Traditional indications for intervention include severe AS in symptomatic patients, and in the presence of LV dysfunction (LV ejection fraction [LVEF] <50%) or concomitant cardiac surgery. Additional indications encompass severe AS in asymptomatic patients with low surgical risk when the serum B-type natriuretic peptide levels are >3 times normal, when testing shows an increase in aortic velocity ≥0.3 m/s per year, or when there is a progressive decline in LVEF to <60% on at least 3 serial imaging studies.^7^

Moreover, 2 new categories of patients are now being considered for early intervention: asymptomatic patients with severe AS and symptomatic patients with moderate AS. These indications are supported by 2 recently published randomized clinical trials. In the RECOVERY trial, early surgical intervention in asymptomatic patients with very severe AS demonstrated superior outcomes in terms of 30-day mortality or cardiovascular mortality at a median follow-up of approximately 6 years. This study showed a cardiovascular mortality of 1%, compared to 15% in patients who underwent surgery versus those who received conservative treatment, with a number needed to treat of 20 patients to prevent 1 cardiovascular death within 4 years.^8^ The AVATAR trial showed that in asymptomatic patients with severe AS and normal LVEF, early SAVR significantly reduced the primary composite endpoint (all-cause death, acute myocardial infarction, stroke, and unplanned heart failure hospitalization) compared to conservative strategy (hazard ratio [HR], 0.46; 95% confidence interval [CI], 0.23-0.90; *P* = .02).^9^

## Lifetime Management of AS

Effective AS management requires a comprehensive approach that focuses on lifetime management that involves balancing the patient's life expectancy with valve durability while anticipating potential risks and the need for future reintervention. For younger patients, particularly those age ≤50 years, the decision often favors surgical options that offer longer-lasting results. In these cases, the use of mechanical aortic valves is frequently recommended because of their durability. The Ross procedure is also an attractive and feasible alternative in experienced centers.

### Ross Procedure

The Ross procedure involves replacing the aortic valve with the patient's own pulmonary valve and placing a pulmonary valve homograft in the pulmonary position.^10^ Advantages of the Ross procedure include avoiding the need for a prosthetic heart valve, reducing the risks associated with anticoagulation, and providing excellent valve hemodynamics. Some studies have shown a survival rate of >80% up to 20 years after surgery.^10^ However, both the pulmonary homograft and the neo-aortic valve (the replaced pulmonary valve) are at risk of degeneration over time. This complex procedure should be reserved for younger patients with suitable anatomy and tissue characteristics, particularly when anticoagulation is contraindicated or undesirable, and should be performed only at comprehensive valve centers by experienced surgeons.^7^

### Decision Between Mechanical Versus Biological Aortic Valve Prostheses

Mechanical valves are known for their exceptional durability, with a reoperation rate significantly lower than that of tissue valves—approximately 0.5% per patient per year. The primary challenge associated with mechanical valves is the need for lifelong anticoagulation. However, recent advances such as the On-X valve have allowed for lower International Normalized Ratio targets, between 1.5 and 2.0.^11^ This reduction in the International Normalized Ratio requirement potentially broadens the suitability of mechanical valves for a larger patient population by minimizing the risks associated with anticoagulation.

In contrast, bioprostheses do not require lifelong anticoagulation but are susceptible to structural and nonstructural valve degeneration. This risk is particularly heightened in patients with risk factors for earlier valve failure, such as younger age, female sex, small annulus size, atrial fibrillation, diabetes, metabolic syndrome, hypercholesterolemia, hypertension, end-stage renal disease, hyperparathyroidism, and pregnancy.

## Decision Between TAVR versus SAVR

### Clinical and Anatomic Considerations

Current American College of Cardiology and European Society of Cardiology/European Association for Cardio-Thoracic Surgery guidelines recommend TAVR as the preferred treatment option for older patients with a life expectancy >1 year or those considered at high surgical risk (Society of Thoracic Surgeons Predicted Risk of Mortality or EuroSCORE II >8%) (Table 1).^7^^,^^12^ Additional factors favoring TAVR include advanced age, severe frailty, previous cardiac surgery (particularly with intact coronary artery bypass grafts at risk of injury during repeat sternotomy), sequelae of chest radiation, porcelain aorta, high likelihood of severe patient–prosthesis mismatch (PPM), and feasibility of the transfemoral approach. The definition of a feasible transfemoral approach depends on the THV platform but generally requires a common femoral artery diameter >5 to 5.5 cm, the absence of hostile aorto-iliac-femoral anatomy (eg, extensive and circumferential calcification, significant atherosclerosis, or tortuosity), and no previously implanted arterial grafts.

**Table 1:** AHA/ACC- and ESC/EACTS-recommended treatment strategies for severe aortic stenosis

Guideline	TAVR	SAVR	Heart team discussion
2020 AHA/ACC guideline	Patients age 80 y	Patients age <65 y who are candidates for intervention	Patients age 65 to 80 y and appropriate vascular anatomy for transfemoral TAVR
2021 ESC/EACTS guideline	Patients age >75 y, high surgical risk, and suitable for transfemoral TAVR	Patients age <75 y, low surgical risk, and unsuitable for transfemoral TAVR	Every other patient

*AHA*, American Heart Association; *ACC*, American College of Cardiology; *ESC*, European Society of Cardiology; *EACTS*, European Association for Cardio-Thoracic Surgery; *TAVR*, transcatheter aortic valve replacement; *SAVR*, surgical aortic valve replacement.

In contrast, factors favoring SAVR include lower surgical risk, younger age, longer life expectancy, active or suspected endocarditis, challenging or impossible transfemoral access, aortic annular dimensions unsuitable for TAVR devices, valve morphology unfavorable for TAVR (eg, high risk of coronary obstruction, heavy leaflet/left ventricle outflow tract calcification), bicuspid aortic valve, thrombus in the aorta or left ventricle, and the presence of concomitant cardiac conditions requiring intervention).^7^

### Age Considerations

Regarding age, while the American College of Cardiology guidelines suggests TAVR for patients age >65 years, the European Society of Cardiology/European Association for Cardio-Thoracic Surgery guidelines are more conservative, recommending TAVR for those age ≥75 years. This difference stems from the mean age observed in 2 major randomized clinical trials evaluating TAVR in low-risk patients: 73 years in the PARTNER 3 trial and 74 years in the Evolut Low-Risk trial.^5^^,^^13^ Therefore, the final decision should consider all relevant factors discussed above, including heart team recommendations and the preferences of the patient and their family.

### Valve Anatomy Considerations

The presence of a bicuspid aortic valve significantly influences treatment decisions, especially when combined with a high calcium burden. Although registry data have suggested similar outcomes between bicuspid and tricuspid valves, previous randomized clinical trials^5^^,^^13^ excluded bicuspid aortic valves owing to concerns about potential complications. The NOTION-2 trial was the first to include patients with bicuspid valve anatomy (49 patients in the TAVR group vs 51 in the SAVR group).^14^ One-year outcomes from NOTION-2 showed no significant difference between TAVR and SAVR in terms of death, stroke, or rehospitalization for tricuspid patients (8.7% vs 8.3%; HR, 1.0; 95% CI, 0.5-2.3). However, SAVR was found to be superior for bicuspid patients (14.3% vs 3.9%; HR, 3.8; 95% CI, 0.8-18.5). Long-term follow-up of this study will provide further insights into which types of bicuspid anatomy may be suitable for transcatheter intervention without compromising outcomes.

### Trends in TAVR and SAVR

**Figure 1:**
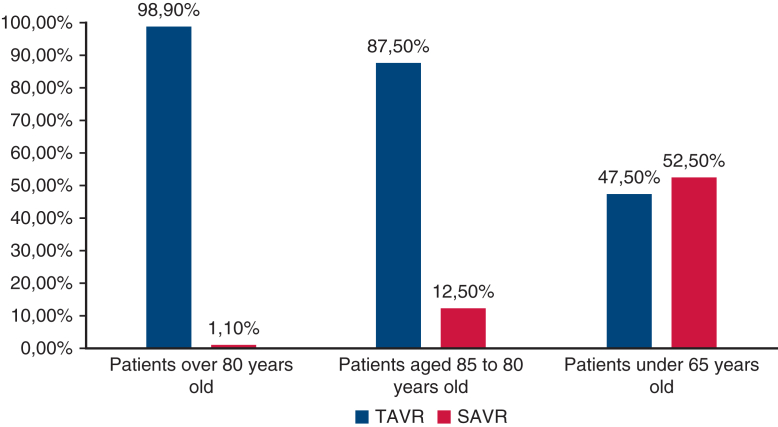
2022 US SAVR/TAVR data distributed by age group. *SAVR*, Surgical aortic valve replacement; *TAVR*, transcatheter aortic valve replacement.

The annual number of TAVR procedures exceeded that of isolated SAVR in the United States in 2015. By 2018, TAVR surpassed the combined total of all types of SAVR procedures.^15^ Currently, TAVRs account for nearly one-half of all AS interventions performed in the United States for patients age <65 years (Figure 1)^16^—a trend that not only reflects advancements in technology and growing confidence in TAVR, but also signals a significant shift in clinical practice.

## Long-Term Goals

### Prosthesis Durability

**Figure 2:**
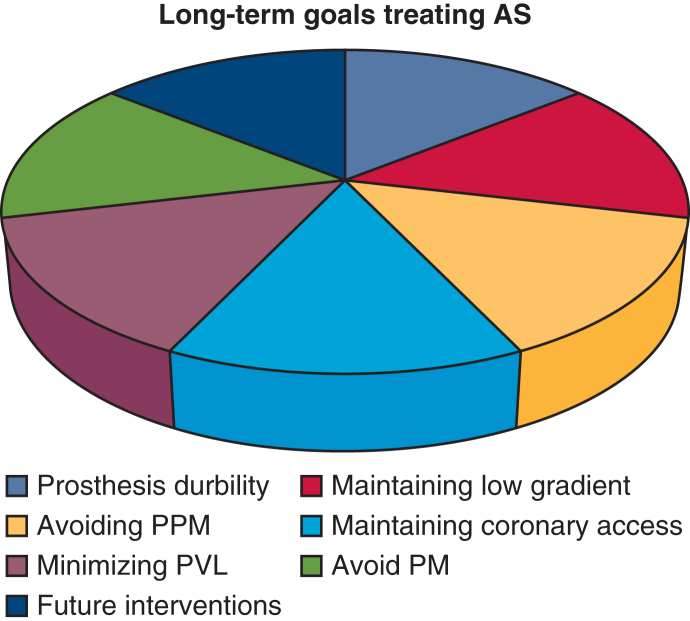
Key goals in AS management of aortic stenosis. *PM*, Pacemaker; *PPM*, patient–prosthesis mismatch; *PVL*, paravalvular leak.

Assessing the long-term durability of valve prostheses for biological SAVR and TAVR remains challenging (Figure 2). Surgical data on long-term outcomes shows significant variability owing to differing definitions of structural valve degeneration and the lack of systematically collected core laboratory data.^17^ This inconsistency complicates drawing reliable conclusions about the lifespan of these prostheses. Recent findings from the PARTNER 3 trial, published in 2023, provide insight into the durability of these valve types. The trial reported rates of stage 2/3 structural valve degeneration of 4.2% for TAVR and 3.8% for SAVR over a period of up to 5 years. Additionally, the reoperation rate for valve-related issues was 2.6% for TAVR and 3.0% for SAVR during the same period.^4^ These data suggest comparable performance of the 2 modalities in the medium term, although the true long-term outcomes remain incompletely understood. Furthermore, the Evolut Low-Risk trial, which tracked outcomes for up to 4 years, highlighted the performance benefits of TAVR, showing a consistently larger effective orifice area and a lower mean gradient compared to some surgical options.^18^

Some SAVR and TAVR outcomes that should be pursued, as they will impact both short- and long-term valve performance, are discussed below.

### Bioprosthesis Gradients

Ensuring that the valve maintains a low transvalvular pressure gradient and avoiding PPM, is essential for optimal heart function. For patients with a small aortic annulus undergoing SAVR, aortic root enlargement and the use of rapid deployment valves are viable options to optimize hemodynamic performance by allowing for the implantation of a larger valve. In contrast, the size of the root cannot be modified in TAVR. However, the use of self-expandable valves offers a practical solution for patients with a small annulus, as they conform better to the native annulus geometry than their balloon-expandable counterparts, which helps achieve a larger effective orifice area, resulting in a lower pressure gradient.^19^

### Coronary Artery Access

Maintaining or ensuring coronary access is essential for future coronary interventions, particularly in younger patients and those with concomitant coronary artery disease. During SAVR, achieving precise alignment of the valve commissures is more straightforward, significantly enhancing postoperative coronary access. The anatomical configuration of surgical valves typically facilitates unimpeded access to the coronary ostia. In contrast, coronary access post-TAVR presents unique challenges, particularly due to factors such as commissural misalignment, supra-annular leaflet, and tall stent frame design.

### Paravalvular Leak

Minimizing the risk of paravalvular leak (PVL) is crucial, as it can impact valve efficacy and lead to adverse outcomes.^20^ Initially, PVL rates were considerably higher in TAVR compared to SAVR, where PVL rates typically range from 0% to 5%. This disparity has been linked to worse outcomes in patients experiencing any degree of PVL.^20^ However, technological advancements in TAVR devices, including design modifications such as the incorporation of an outer skirt on newer valve models, have led to a significantly reduced incidence of PVL.^5^^,^^21^

### New Conduction Disturbances and Need for Permanent Pacemaker Implantation

Avoiding the need for permanent pacemaker implantation (PPI) post-TAVR is desirable, because it is associated with increased morbidity. Recent meta-analyses have linked PPI to an increased risk of all-cause mortality and heart failure rehospitalization.^22^ Despite advancements aimed at reducing PPI post-TAVR, such as the cusp overlap technique, the Evolut Low-Risk trial reported a 23.8% incidence of PPI within 4 years after TAVR.^5^

### Feasibility of Future Reinterventions

Planning for future interventions, such as valve-in-valve and redo TAVR, is essential if the initial prosthetic valve fails. Although valve-in-valve TAVR is generally considered safe and straightforward, it is not without risks, particularly the risk of coronary obstruction, which is significantly greater compared to that with TAVR in native valves.^23^ The reported rates of bioprosthesis reintervention are approximately 7% at 10 years and 15% at 20 years, with some substantial differences depending on the patient's age and the design, generation, and model of the implanted bioprosthetic valve. The main cause of reintervention is irreversible stage 3 hemodynamic valve deterioration, leading to bioprosthetic valve dysfunction.^17^

Redo TAVR requires careful consideration owing to the risk of coronary obstruction, which varies according to the THV configuration—whether a short balloon-expandable valve or tall self-expandable valve. Typically, the “tall-in-tall” combination carries the highest risk because of sinus sequestration, while a short valve within another short valve (short-in-short) presents the lowest risk. The preferred approach to treating a failed self-expandable valve is often with a balloon-expandable valve, and vice versa. This strategy helps minimize coronary obstruction risk and maintain optimal hemodynamic performance.

If coronary obstruction remains a significant concern and leaflet modification techniques such as BASILICA (which involves creating a split in the leaflet to facilitate coronary access) are infeasible or fail, surgical options may be necessary. In the case of a TAVR explantation, especially if many years have passed since the index TAVR, the embedded stent frame may become endothelialized, complicating its removal. An alternative surgical approach involves removing the valve leaflets and performing another TAVR under direct vision or resorting to more extensive aortic root surgery.^24^

Planning a TAV-in-TAV procedure is done using the same computed tomography angiography used to plan any TAVR procedure. The semiautomated software 3mensio Valves has a critical role in simulating the TAVR-in-TAVR and predicting the risk of coronary obstruction due to direct obstruction or sinus sequestration. Additionally, a recent app created by Bapat and colleagues (Redo TAV) can be helpful when performing this type of procedure, as information added to the app helps anticipate the risk of coronary obstruction and other potential procedural challenges.

## Conclusions

The management of AS has undergone significant transformations with the advent of advanced diagnostic modalities and therapeutic options, shifting toward less-invasive transcatheter procedures and refined surgical techniques. Innovations in valve technology and procedural approaches have improved outcomes, reducing complications and enhancing the durability of valve prostheses. Contemporary guidelines now emphasize a more personalized approach to treatment, considering patient-specific factors such as age, anatomic suitability, and individual risk profiles. This patient-centric strategy, coupled with emerging evidence from recent clinical trials, supports earlier intervention in selected patient populations, potentially improving long-term outcomes and quality of life. As the field continues to evolve with ongoing research and technological advancements, the management of aortic stenosis is poised to become even more precise, offering patients safer, more effective, and durable treatment alternatives.

## Conflict of Interest Statement

Dr Tagliari reports speaking fees from Biotronik, Boston Scientific, and Meril Life. Dr Tang reports receiving speaker's honoraria and serving as a physician proctor, consultant, advisory board member, TAVR publications committee member, APOLLO trial screening committee member, and IMPACT MR steering committee member for Medtronic; receiving speaker's honoraria and serving as a physician proctor, consultant, advisory board member and TRILUMINATE trial anatomic eligibility and publications committee member for Abbott Structural Heart; serving as an advisory board member for Boston Scientific and JenaValve; serving as a consultant and physician screening committee member for Shockwave Medical; serving as a consultant for NeoChord, Peija Medical, and Shenqi Medical Technology; and receiving speaker's honoraria from Siemens Healthineers. All other authors reported no conflicts of interest.

The *Journal* policy requires editors and reviewers to disclose conflicts of interest and to decline handling or reviewing manuscripts for which they may have a conflict of interest. The editors and reviewers of this article have no conflicts of interest.

## References

[1] Ghoreishi M., Thourani V.H., Badhwar V. (2021). Less-invasive aortic valve replacement: trends and outcomes from the Society of Thoracic Surgeons Database. Ann Thorac Surg.

[2] Martinsson A., Rasalingam R., Jeppsson A. (2024). Stable incidence and outcomes for patients with incident aortic stenosis: how can we translate excellent trial data into improved clinical outcomes in the population?. Eur Heart J.

[3] Barker C.M., Reardon M.J. (2014). The CoreValve US pivotal trial. Semin Thorac Cardiovasc Surg.

[4] Mack M.J., Leon M.B., Thourani V.H. (2023). Transcatheter aortic-valve replacement in low-risk patients at five years. N Engl J Med.

[5] Popma J.J., Deeb G.M., Yakubov S.J. (2019). Transcatheter aortic-valve replacement with a self-expanding valve in low-risk patients. N Engl J Med.

[6] Everett R.J., Clavel M.A., Pibarot P., Dweck M.R. (2018). Timing of intervention in aortic stenosis: a review of current and future strategies. Heart.

[7] Beyersdorf F., Vahanian A., Milojevic M. (2021). 2021 ESC/EACTS guidelines for the management of valvular heart disease: developed by the task force for the management of valvular heart disease of the European Society of Cardiology (ESC) and the European Association for Cardio-Thoracic Surgery (EACTS). Eur J Cardiothorac Surg.

[8] Kang D.H., Park S.J., Lee S.A. (2020). Early surgery or conservative care for asymptomatic aortic stenosis. N Engl J Med.

[9] Banovic M., Putnik S., Penicka M. (2022). Aortic valve replacement versus conservative treatment in asymptomatic severe aortic stenosis: the AVATAR trial. Circulation.

[10] Ryan W.H., Squiers J.J., Harrington K.B. (2021). Long-term outcomes of the Ross procedure in adults. Ann Cardiothorac Surg.

[11] Bouhout I., El-Hamamsy I. (2019). The Prospective Randomized On-X valve Anticoagulation Clinical Trial (PROACT): lower is better, but is it good enough?. Glob Cardiol Sci Pract.

[12] Otto C.M., Nishimura R.A., Bonow R.O. (2021). 2020 ACC/AHA guideline for the management of patients with valvular heart disease: a report of the American College of Cardiology/American Heart Association Joint Committee on Clinical Practice Guidelines. Circulation.

[13] Mack M.J., Leon M.B., Thourani V.H. (2019). Transcatheter aortic-valve replacement with a balloon-expandable valve in low-risk patients. N Engl J Med.

[14] Jørgensen T.H., Thyregod H.G.H., Savontaus M. (2024). Transcatheter aortic valve implantation in low-risk tricuspid or bicuspid aortic stenosis: the NOTION-2 trial. Eur Heart J.

[15] Carroll J.D., Mack M.J., Vemulapalli S. (2021). STS-ACC TVT registry of transcatheter aortic valve replacement. Ann Thorac Surg.

[16] Sharma T., Krishnan A.M., Lahoud R., Polomsky M., Dauerman H.L. (2022). National trends in TAVR and SAVR for patients with severe isolated aortic stenosis. J Am Coll Cardiol.

[17] Ternacle J., Hecht S., Eltchaninoff H. (2024). Durability of transcatheter aortic valve implantation. EuroIntervention.

[18] Forrest J.K., Deeb G.M., Yakubov S.J. (2023). 4-year outcomes of patients with aortic stenosis in the evolut low-risk trial. J Am Coll Cardiol.

[19] Herrmann H.C., Abdel-Wahab M., Attizzani G.F. (2022). Rationale and design of the SMall annuli randomized to Evolut or SAPIEN trial (SMART trial). Am Heart J.

[20] Sá M.P., Jacquemyn X., Van den Eynde J. (2022). Impact of paravalvular leak on outcomes after transcatheter aortic valve implantation: meta-analysis of Kaplan-Meier-derived individual patient data. Struct Heart.

[21] Kolte D., Vlahakes G.J., Palacios I.F. (2019). Transcatheter versus surgical aortic valve replacement in low-risk patients. J Am Coll Cardiol.

[22] Sá M.P., Jacquemyn X., Sun T. (2022). Late outcomes of permanent pacemaker implantation after TAVR: meta-analysis of reconstructed time-to-event data. J Soc Cardiovasc Angiogr Interv.

[23] Edelman J.J., Khan J.M., Rogers T. (2019). Valve-in-valve TAVR: state-of-the-art review. Innovations (Phila).

[24] Tang G.H.L., Zaid S., Kleiman N.S. (2023). Explant vs redo-TAVR after transcatheter valve failure: mid-term outcomes from the EXPLANTORREDO-TAVR International Registry. JACC Cardiovasc Interv.

